# Frequency and risk factors of low birth weight in term pregnancy

**DOI:** 10.12669/pjms.321.8120

**Published:** 2016

**Authors:** Ayesha Khan, Farah Deeba Nasrullah, Riffat Jaleel

**Affiliations:** 1 Prof. Ayesha Khan, FRCOG, Chairperson of Obstetrics and Gynaecology Department, Department of Obstetrics and Gynaecology, Dow University of Health Sciences and Civil Hospital, Karachi, Pakistan; 2 Dr. Farah Deeba Nasrullah, FCPS, Assistant Professor of Obstetrics and Gynaecology, Department of Obstetrics and Gynaecology, Dow University of Health Sciences and Civil Hospital, Karachi, Pakistan; 3 Dr. Riffat Jaleel, FCPS, Associate Professor of Obstetrics and Gynaecology, Department of Obstetrics and Gynaecology, Dow University of Health Sciences and Civil Hospital, Karachi, Pakistan

**Keywords:** Low birth weight, High risk factor

## Abstract

**Objective:**

To determine frequency of Low Birth Weight (LBW) at term and to determine frequency of various associated risk factors.

**Methods:**

This cross-sectional study was conducted in Department of Obstetrics and Gynaecology Layari General Hospital and Dow University of Health Sciences Karachi between January 2007 to July 2008. Women with singleton pregnancy of 37 and above completed weeks were identified. Those women who gave birth to babies with less than 2500gm were recruited in the study. In all those patients who gave birth to LBW babies risk factors were assessed. The variables including age, parity, booking status, maternal weight and height, socioeconomic condition, smoking, fetal gender and birth weight were noted on a questionare. The data was analyzed on SPSS Version 15. Frequency of LBW and its associated risk factors were determined.

**Results:**

During study period 10.6% patients delivered LBW babies. Antenatal care was not received in 67% patients. Parity was less than three in 87%. In 26% of patients maternal weight was less than 50 kg and in 37% patients with LBW, maternal height was less than five feet. Majority of patients were anaemic (72%) including 20% with haemoglobin < 7 grams.

**Conclusion:**

LBW is associated with a group of factors which may be regarded as high risk factors. These include low socio economic status, anaemia, primiparity, short maternal height and less than average weight.

## INTRODUCTION

Weight of a baby at term depends on gestational age and rate of fetal growth in uterus. Babies born may be appropriate for gestational age but are small because of preterm delivery. Babies who are small for gestational age may be born preterm or term. A baby is said to be small for gestational age when the gender specific birth weight is below the 10^th^ percentile for the appropriate gestational age. More than 70% of these Low Birth Weight (LBW) babies are small due to constitutional and environmental factors. Small for gestational age may be due to pathological reasons when it is called as intra uterine growth restriction.

Depending upon birth weight and gestational age, WHO^1^ categorizes babies in three groups, Small for gestational age, Appropriate for gestational age and Large for gestational age. Depending upon these criteria, LBW baby is defined as baby having a weight less than 2.5kg within 24 hours of birth. This group of babies has contribution in high perinatal mortality and morbidity. This high risk is due to the fact that these babies are at higher risk of infectious diseases, malnutrition and growth failure. They are also at risk of suffering from abnormal cognitive development, neurological impairment and poor school performance. In adult life they are at greater risk of diseases like hypertension and diabetes.

In developing countries, LBW is a major determinant of perinatal mortality and morbidity. Across the world Neonatal mortality is 20 times more likely for LBW babies as compared to babies heavier than 2.5kg.^2^ It is estimated that 72% of LBW infants in developing countries are born in Asia.^2^ In Pakistan reported prevalence of LBW is 12-25%.^3^

An improvement with increment in the birth weight of babies is expected to reduce the occurrence of LBW babies; therefore this study was conducted with the intention to identify risk factors present among mothers who delivered babies with LBW. Identification of factors which carry risk of LBW may help to attempt their correction and counseling when possible. This modification will help in decreasing perinatal mortality and morbidity.

## METHODS

This cross sectional study was conducted in Department of Obstetrics and Gynaecology, Dow Medical College & Layari General Hospital Karachi, from January, 2007 to July 2008. The selection criteria were singleton pregnancy with ≥37 completed weeks of gestation. Patients with multiple pregnancy, in-utero death, fetal abnormality, medical disorders like hypertension, diabetes, renal disease and cardio-respiratory disease, antepartum haemorrhage and those who delivered preterm were excluded. All those patients who delivered during the study period and met the inclusion criteria were recruited in the study by designated post graduate present in labor ward. An informed consent was taken from all selected patients. Variables including age, parity, maternal weight and height, family income, haemoblobin (Hb), birth weight, fetal sex were noted on predesigned questionare.

After delivery all babies were weighed on scale in labour room after clamping and cutting the umbilical cord, within one hour of birth. Babies were termed as normal weight if birth weight was 2.5 to 4 kg, LBW if less than 2.5 kg and macrosomic if more than 4 kg. Anemia was classified as no anemia if Hb was more than 10 g/dl, mild to moderate if Hb was 7 – 10 g/dl and severe anemia if Hb was less than 7 g/dl.

The data were analyzed using SPSS version15. Mean ± SD was calculated for numerical variables like age, parity and gestational age. Frequencies and percentiles were calculated for categorical variables such as birth weight, maternal age, height, weight, parity, booking status, monthly income, anaemia, smoking and fetal gender.

## RESULTS

The study included 947 singleton live births at term. Birth weight was less than 2.5kg in 100 patients. Mean age of patients was 26 years. Majority of patients (82%) were 20-30 years old (Table-I). In patients who gave birth to babies less than 2.5 kg, majority of patients (67%) were non booked. Mean gestational age at time of delivery was 38 weeks. A large number of patients (87%) had parity less than 3 including 54% primigravida. Average weight of babies was 2915gm. The frequency of LBW was 10.6% (Table-II). Risk factors in LBW group are shown in Table-III. Maternal anaemia was significantly seen in LBW patients (72%). Maternal haemoglobin was less than 7 in 20% patients. Only 5% patients were smokers. Majority of patients with LBW belonged to low income group (91%). Maternal height and weight were also found as significant risk factors. Maternal height was< 5 feet in 37% and weight was < 50 kg in 26% patients. There was predominance of female gender (54%) in LBW group.

**Table-I:** Demographic features.

Demographic features	N	Minimum	Maximum	Mean	Std. Deviation
Age of patients	947	16	45	26 years	±5.100
Parity of patient	947	0	9	3	±1.987
Gestational age	947	37	42	38 weeks	±1.225

**Table-II:** Birth weight of baby.

	Frequency	Percent	Cumulative Percent
Average weight	809	85.4	85.4
Low birth weight	100	10.6	96.0
Macrosomia	38	4.0	100.0

Total	947	100.0	

**Table-III:** Risk factors in patients with LBW.

Risk Factors		No. of patients &%
Age of patients	20-30 years	82%
	>30 years	12%
	< 20 years	6%
Parity	Primigraviga	54%
	Para 1-3	33%
	Para> 3	13%
Antenatal care	Non booked	67%
	Booked	33%
Monthly income	< 5000/month	34%
	5000-10000/m	57%
	>10000/m	9%
Anaemia	Normal Hb	28%
	Mild- moderate anaemia	52%
	Severe anaemia	20%
Maternal height	< 5 feet	37%
	5- 5.2 feet	58%
	>5.2 feet	5%
Maternal weight	< 50kg	26%
	50-55kg	70%
	>55 kg	4%
Smoking	Smokers	5%
	Non-smokers	95%
Fetal gender	Female	54%
	Male	46%

## DISCUSSION

Weight of baby reflects the maternal health and nutritional status of mother before conception and during pregnancy. Growth of the baby in utero is determined by maternal, fetal and placental factors.

A baby’s birth weight is related to birth weight of both parents and more strongly through the maternal line.^4^ Women born with LBW have a higher risk of also having LBW babies. When these female babies enter motherhood they are at risk of having hypertension, diabetes and delivering babies with LBW. The intergenerational transmission of birth weight and its delayed effects later in life are a matter of concern.

In this study risk factors which are strongly associated with LBW babies are identified as primiparity, low socioeconomic status, anemia, maternal height and weight. In a meta analysis a significant association is noted between abuse and low birth weight.^5^ Abuse during pregnancy is associated with low socioeconomic status, poor maternal weight gain, anaemia, unhealthy diet and pshycological morbidity.

The adverse effects of maternal smoking for human pregnancy are well known. Use of smoking and illicit drugs during pregnancy is associated with pregnancy complication and Low birth weight. Maternal smoking reduces mean birth weight by about 150-200 gm and doubles the risk of LBW associated with restriction of intra uterine growth. Moreover a dose response effect has been described with occurrence of low birth weight increasing with the number of cigarettes smoked per day. The risk of LBW was reported 4.1 times high in women who are addicted to any tobacco product as compared to those who were not exposed to any tobacco. Not only cigarette smoking, tobacco chewing is also a risk factor for LBW.^6^ In contrast to these reports only 5% of women in current study gave history of smoking.

In this cross sectional study the frequency of LBW was found as 10.6%. This is comparable to the figure of 12.7% reported earlier.^1^ It is lower than previous report of an incidence of 25% LBW babies.^3^ The frequency of LBW was reported between 15-30% from South Asian countries.^7^ The proportion of LBW reported from India is 21.5%^8^ to 26.8%.^9^ The National Nutritional Survey data reports the frequency of 12- 25% in Pakistan.^3^

Studies have shown that maternal age is an important predictor for the size of the new born. The relation of age and LBW is reported differently by authors. The incidence of LBW was significantly higher in the group of teen age mothers i.e 65.52%.^10^ Viengsaahone reported young maternal age as significant risk factor for LBW with OR=8.6, 95%CI=2.4-30.7.^11^ The risk of LBW is also reported proportionally related to maternal age. Rizvi et al. reported increased risk of LBW with increasing maternal age.^12^ A study from India failed to find any association between LBW and increasing maternal age as significant risk factors.^8^ In the current study 82% of patients belonged to age group 20 to30 years. This is comparable to study from Jos where 78% of LBW delivered in age group 20 to 35 years.^1^

In the current study primiparity was noted in 54% of patients with LBW. This is consistent with a report from Kozuki who reported nulliparity and para above 3 at risk of having LBW babies.^13^

Modest degree of malnutrition has effect on birth weight of baby. Mother with optimal weight are considered to have babies of average weight. Increased maternal birth weight and pre pregnancy maternal over weight or obesity had a strong co relation with new born high birth weight. A study from New Zealand reports mothers of LBW were shorter and reported lower pre pregnancy weight as compare to mothers of AGA infants.^14^

In this study, 26% of the mothers of low birth babies were less than 50 kg weight and 70% were between 50-54 kg. Zhen Han reported underweight women had an increased risk of LBW babies RR 1.64, 95% CI1.38—1.94.^15^

There is increased risk of LBW of newborn in women of short height.^16^ This coincides with current study in which 37% mothers were short with height less than 5 feet and 58% had height 5-5.2. feet.

Lack of ANC was reported as significantly associated with LBW. Lin-Lin–Dal suggested number of antenatal visits had a U trend effect on LBW. He reported increasing number of prenatal visits decrease risk of LBW.^17^ Kotelchuck^18^ reported a relative risk of LBW as 1.47 in patients with inadequate antenatal visits and risk of 0.56 in women with adequate ante natal visits. Provision of ANC is expected to reduce the risk of LBW. It creates health awareness and timely identification of complications.

From India Mumbare reported 62.4% mothers who delivered LBW babies did not receive antenatal care.^8^ This is consistent with the findings in the current study in which 67% mothers of LWB babies failed to seek antenatal care.

Severe maternal starvation during pregnancy has a major impact on fetal growth. During winter 1944-1945 Dutch population suffered severe famine and mean maternal caloric intake fell to 450-750 Kcal a day with subsequent decrease of 250 gm in baby’s birth weight. Kayode has reported a high incidence of LBW babies among women living in rural areas with low coverage of safe water supply. This could be because of increased episodes of gastro intestinal infections impairing normal fetal development.^19^ Poverty resulting in poor nutritional status contributes to the occurrence of LBW. In the studied population 34% of mothers who delivered LBW babies belonged to low socioeconomic class with a monthly income of less than Rs. 5000 and further 57% had monthly income between Rs. 5000-10,000. This is close to the figures coated by Sharma i.e 28.9% having income less than Rs. 2000 and 42.9% having income between Rs. 2000 to 7000 plus.^20^

Anaemia is the commonest medical disorder in pregnancy. 56% of pregnant women in developing countries are anaemic. Risk of anaemia in Pakistan ranges from 8-33%. Anaemic mothers tend to deliver small babies. A strong relationship exists between maternal anaemia and LBW babies. Badshah has reported a significantly higher incidence of LBW babies among anaemic as compare to non anaemic mothers.^21^

Lone, et al.^22^ reported risk of LBW babies in anaemic population as 1.9 time higher in Pakistan. Ahmed, et al.^23^ reported an association of maternal anaemia with increased risk of LBW. Similar findings were also reported in other studies from Pakistan.^24^ There is strong relationship between anaemia and LBW. In current study 72% of mothers with LBW were anemic including 20% having severe anaemia. Anaemia is a problem which can be prevented. Correction of anaemia is expected to result in less frequency of LBW and is likely to lower PNM in population. In Pakistan, iron deficiency is the commonest cause of anaemia in pregnancy due to low socioeconomic load, malnutrition and multiparity. Iron supplements during pregnancy is expected to decrease the risk of anaemia and to protect the babies against LBW. This is also reported in studies from America.^25^

## CONCLUSION

There is an identifiable group of women with high risk for LBW. Low socioeconomic status, anaemia, maternal height and weight are significantly associated with LBW. Correction of anaemia, intake of a balanced diet, utilization of antenatal care is expected to reduce the frequency of LBW babies and decrease perinatal mortality.
